# Medicaid policy data for evaluating eligibility and programmatic changes

**DOI:** 10.1186/s13104-023-06525-6

**Published:** 2023-10-03

**Authors:** Paul R. Shafer, Amanda Katchmar, Steven Callori, Raisa Alam, Roshni Patel, Sugy Choi, Samantha Auty

**Affiliations:** 1 Department of Health Law, Policy, and Management School of Public Health, Boston University, 715 Albany Street, Boston, MA 02118 USA; 2 Alix School of Medicine, Mayo Clinic, 200 1st Street SW, Rochester, MN 55905 USA; 3 Health Management Associates, 31 Saint James Avenue Suite 920, Boston, MA 02116 USA; 4 James E. Rogers College of Law, University of Arizona, 1201 East Speedway Boulevard, Tucson, AZ 85721 USA; 5 Department of Population Health, Grossman School of Medicine, New York University, 180 Madison Avenue, New York, NY 10016 USA

**Keywords:** Medicaid, Eligibility, Enrollment, Renewal, Administrative burdens, Premiums, Cost-sharing, Managed care, Policy, Equity

## Abstract

**Objectives:**

Medicaid and the Children’s Health Insurance Program (CHIP) provide health insurance coverage to more than 90 million Americans as of early 2023. There is substantial variation in eligibility criteria, application procedures, premiums, and other programmatic characteristics across states and over time. Analyzing changes in Medicaid policies is important for state and federal agencies and other stakeholders, but such analysis requires data on historical programmatic characteristics that are often not available in a form ready for quantitative analysis. Our objective is to fill this gap by synthesizing existing qualitative policy data to create a new data resource that facilitates Medicaid policy research.

**Data description:**

Our source data were the 50-state surveys of Medicaid and CHIP eligibility, enrollment, and cost-sharing policies, and budgets conducted near annually by KFF since 2000, which we coded through 2020. These reports are a rich source of point-in-time information but not operationalized for quantitative analysis. Through a review of the measures captured in the KFF surveys, we developed five Medicaid policy domains with 122 measures in total, each coded by state-quarter—1) eligibility (28 measures), 2) enrollment and renewal processes (39 measures), 3) premiums (16 measures), 4) cost-sharing (26 measures), and 5) managed care (13 measures).

## Objective

Medicaid and the Children’s Health Insurance Program (CHIP) provide health insurance coverage to more than 90 million Americans as of early 2023 [1]. There is substantial variation in eligibility criteria, application procedures, premiums, and other programmatic characteristics across states and over time. States use waiver authority provided in the Social Security Act to propose wide-ranging demonstration projects to reshape their programs [2]. Programmatic differences between state Medicaid programs may influence Medicaid enrollment, access to health care, and downstream health outcomes. Indeed, considerable cross-state variation in eligibility and administrative burdens to get and remain enrolled have often yielded inequities in participation and outcomes within the program [3–5]. Yet, the Government Accountability Office has noted the lack of rigorous evaluation required by Centers for Medicare and Medicaid Services (CMS) or conducted by states [6].

As such, analysis of Medicaid policy changes is important for state and federal agencies and other stakeholders, and is therefore critical and highly fundable work. However, this requires an understanding of historical eligibility criteria and programmatic characteristics that are not readily available in a form easily used for quantitative analysis. An example of this, published in 2015, used sensitivity to historical Medicaid eligibility changes to predict effects of the 2014 Medicaid expansion on use of care in the Veterans Health Administration, in which the authors noted that it was “based on historical data available only through 2008” [7].

Our objective is to fill this gap by synthesizing existing qualitative policy data to create a new data resource that facilitates Medicaid policy research. These data can be easily combined with health insurance claims, surveys, or other forms of quantitative data. As novel Medicaid claims data have become available from CMS [8], freely accessible Medicaid policy data will be useful for researchers.

## Data description

Our historical source data consists of 50-state surveys by KFF on Medicaid and CHIP eligibility, enrollment, and cost-sharing policies, and Medicaid budgets. These surveys have been conducted annually by KFF since 2000 and were coded by our team through 2020 [9, 10]. These reports are a rich source of point-in-time information, which KFF uses to support its descriptive reports and interactive data visualizations on their website. Our coding entailed parsing text, maps, and tables from these annual reports into quantitative data (e.g., federal poverty level eligibility thresholds by category of Medicaid eligibility – continuous variable; availability of online application – binary variable). These data can supplement gaps in existing Medicaid policy data sets that are more narrowly focused and/or capture different timeframes [11–13].

Our research team developed five policy domains from a thorough review of the KFF surveys, resulting in a total of 122 measures—1) eligibility (28 measures), 2) enrollment and renewal processes (39 measures), 3) premiums (16 measures), 4) cost-sharing (26 measures), and 5) managed care (13 measures). To manage and house the Medicaid policy data, we used MonQcle (https://monqcle.com), a legal epidemiology tool available through the Center for Public Health Law Research at Temple University. Our team used double data entry of each measure with consensus meetings to address any inconsistencies in coding when beginning the project (approximately 4% of records), with the remainder being entered by a single coder and reviewed for accuracy by another. We coded each measure by state-quarter, capturing the timing of policy changes, and created domain-specific Excel, Stata, and SAS longitudinal data sets that researchers can merge on to other data sets (Table 1) [14].

**Table 1:** Overview of data files/data sets

Label	Name of data file/data set	File types (file extension)	Data repository and identifier (DOI or accession number)
Data file 1	elig_codebook	MS Word file (.docx)	Harvard Dataverse (10.7910/DVN/KAYSAB) Shafer P, Katchmar A, Callori S, Alam R, Patel, R, Choi S, Auty S. Medicaid policy data for evaluating eligibility and programmatic changes. Harvard Dataverse. 2023. 10.7910/DVN/KAYSAB.
Data file 2	enr_codebook	MS Word file (.docx)	Harvard Dataverse (10.7910/DVN/KAYSAB) Shafer P, Katchmar A, Callori S, Alam R, Patel, R, Choi S, Auty S. Medicaid policy data for evaluating eligibility and programmatic changes. Harvard Dataverse. 2023. 10.7910/DVN/KAYSAB.
Data file 3	prem_codebook	MS Word file (.docx)	Harvard Dataverse (10.7910/DVN/KAYSAB) Shafer P, Katchmar A, Callori S, Alam R, Patel, R, Choi S, Auty S. Medicaid policy data for evaluating eligibility and programmatic changes. Harvard Dataverse. 2023. 10.7910/DVN/KAYSAB.
Data file 4	cost_codebook	MS Word file (.docx)	Harvard Dataverse (10.7910/DVN/KAYSAB) Shafer P, Katchmar A, Callori S, Alam R, Patel, R, Choi S, Auty S. Medicaid policy data for evaluating eligibility and programmatic changes. Harvard Dataverse. 2023. 10.7910/DVN/KAYSAB.
Data file 5	managed_codebook	MS Word file (.docx)	Harvard Dataverse (10.7910/DVN/KAYSAB) Shafer P, Katchmar A, Callori S, Alam R, Patel, R, Choi S, Auty S. Medicaid policy data for evaluating eligibility and programmatic changes. Harvard Dataverse. 2023. 10.7910/DVN/KAYSAB.
Data set 1	elig_data	MS Excel file (.xlsx)	Harvard Dataverse (10.7910/DVN/KAYSAB) Shafer P, Katchmar A, Callori S, Alam R, Patel, R, Choi S, Auty S. Medicaid policy data for evaluating eligibility and programmatic changes. Harvard Dataverse. 2023. 10.7910/DVN/KAYSAB.
Data set 2	elig_data	Stata file (.dta)	Harvard Dataverse (10.7910/DVN/KAYSAB) Shafer P, Katchmar A, Callori S, Alam R, Patel, R, Choi S, Auty S. Medicaid policy data for evaluating eligibility and programmatic changes. Harvard Dataverse. 2023. 10.7910/DVN/KAYSAB.
Data set 3	elig_data	SAS file (.v8xpt)	Harvard Dataverse (10.7910/DVN/KAYSAB) Shafer P, Katchmar A, Callori S, Alam R, Patel, R, Choi S, Auty S. Medicaid policy data for evaluating eligibility and programmatic changes. Harvard Dataverse. 2023. 10.7910/DVN/KAYSAB.
Data set 4	enr_data	MS Excel file (.xlsx)	Harvard Dataverse (10.7910/DVN/KAYSAB) Shafer P, Katchmar A, Callori S, Alam R, Patel, R, Choi S, Auty S. Medicaid policy data for evaluating eligibility and programmatic changes. Harvard Dataverse. 2023. 10.7910/DVN/KAYSAB.
Data set 5	enr_data	Stata file (.dta)	Harvard Dataverse (10.7910/DVN/KAYSAB) Shafer P, Katchmar A, Callori S, Alam R, Patel, R, Choi S, Auty S. Medicaid policy data for evaluating eligibility and programmatic changes. Harvard Dataverse. 2023. 10.7910/DVN/KAYSAB.
Data set 6	enr_data	SAS file (.v8xpt)	Harvard Dataverse (10.7910/DVN/KAYSAB) Shafer P, Katchmar A, Callori S, Alam R, Patel, R, Choi S, Auty S. Medicaid policy data for evaluating eligibility and programmatic changes. Harvard Dataverse. 2023. 10.7910/DVN/KAYSAB.
Data set 7	prem_data	MS Excel file (.xlsx)	Harvard Dataverse (10.7910/DVN/KAYSAB) Shafer P, Katchmar A, Callori S, Alam R, Patel, R, Choi S, Auty S. Medicaid policy data for evaluating eligibility and programmatic changes. Harvard Dataverse. 2023. 10.7910/DVN/KAYSAB.
Data set 8	prem_data	Stata file (.dta)	Harvard Dataverse (10.7910/DVN/KAYSAB) Shafer P, Katchmar A, Callori S, Alam R, Patel, R, Choi S, Auty S. Medicaid policy data for evaluating eligibility and programmatic changes. Harvard Dataverse. 2023. 10.7910/DVN/KAYSAB.
Data set 9	prem_data	SAS file (.v8xpt)	Harvard Dataverse (10.7910/DVN/KAYSAB) Shafer P, Katchmar A, Callori S, Alam R, Patel, R, Choi S, Auty S. Medicaid policy data for evaluating eligibility and programmatic changes. Harvard Dataverse. 2023. 10.7910/DVN/KAYSAB.
Data set 10	cost_data	MS Excel file (.xlsx)	Harvard Dataverse (10.7910/DVN/KAYSAB) Shafer P, Katchmar A, Callori S, Alam R, Patel, R, Choi S, Auty S. Medicaid policy data for evaluating eligibility and programmatic changes. Harvard Dataverse. 2023. 10.7910/DVN/KAYSAB.
Data set 11	cost_data	Stata file (.dta)	Harvard Dataverse (10.7910/DVN/KAYSAB) Shafer P, Katchmar A, Callori S, Alam R, Patel, R, Choi S, Auty S. Medicaid policy data for evaluating eligibility and programmatic changes. Harvard Dataverse. 2023. 10.7910/DVN/KAYSAB.
Data set 12	cost_data	SAS file (.v8xpt)	Harvard Dataverse (10.7910/DVN/KAYSAB) Shafer P, Katchmar A, Callori S, Alam R, Patel, R, Choi S, Auty S. Medicaid policy data for evaluating eligibility and programmatic changes. Harvard Dataverse. 2023. 10.7910/DVN/KAYSAB.
Data set 13	managed_data	MS Excel file (.xlsx)	Harvard Dataverse (10.7910/DVN/KAYSAB) Shafer P, Katchmar A, Callori S, Alam R, Patel, R, Choi S, Auty S. Medicaid policy data for evaluating eligibility and programmatic changes. Harvard Dataverse. 2023. 10.7910/DVN/KAYSAB.
Data set 14	managed_data	Stata file (.dta)	Harvard Dataverse (10.7910/DVN/KAYSAB) Shafer P, Katchmar A, Callori S, Alam R, Patel, R, Choi S, Auty S. Medicaid policy data for evaluating eligibility and programmatic changes. Harvard Dataverse. 2023. 10.7910/DVN/KAYSAB.
Data set 15	managed_data	SAS file (.v8xpt)	Harvard Dataverse (10.7910/DVN/KAYSAB) Shafer P, Katchmar A, Callori S, Alam R, Patel, R, Choi S, Auty S. Medicaid policy data for evaluating eligibility and programmatic changes. Harvard Dataverse. 2023. 10.7910/DVN/KAYSAB.

## Limitations

Our measures are not exhaustive of all policies represented in the historical source data or of all Medicaid policies that may warrant evaluation. Our measure set and coded data were limited by what was captured in the historical source data available. Due to coding by year and quarter based on the KFF surveys, specific timing of policy passage and/or implementation may differ slightly from how they are captured in our data. Our measures were either double entered, or coded from the source data and reviewed by another coder for concordance; however, there still may be errors. The accuracy and consistency of our data also depend on the reliability of the information provided in the source data. Not all measures were available, or applicable, for all states and/or years.

## Data Availability

The data described in this Data note can be freely and openly accessed on Harvard Dataverse under 10.7910/DVN/KAYSAB. Please see Table 1 and reference [14] for details and links to the data.

## Abbreviations

CHIP: Children’s Health Insurance Program
CMS: Centers for Medicare and Medicaid Services
